# Explainable multi-modal deep learning for transparent cancer diagnosis: integrating radiology, clinical features, and decision visualization

**DOI:** 10.3389/frai.2026.1767612

**Published:** 2026-02-23

**Authors:** Sital Dash, Laxmi Bewoor, Yashwant Dongre, Amol Bhosle, Kailas Patil, Shrikant Jadhav, Banani Mohapatra, Bhavnish Walia

**Affiliations:** 1 Department of Computer Engineering, Vishakarma University, Pune, Maharashtra, India; 2 Department of Computer Engineering, Vishwakarma Institute of Technology, Pune, Maharashtra, India; 3 Department of Computer Science and Engineering, School of Computing, MIT Art, Design and Technology University, Pune, Maharashtra, India; 4 San Jose State University, San Jose, CA, United States; 5 Walmart, Sunnyvale, CA, United States; 6 Amazon, New York, NY, United States

**Keywords:** attention-based fusion, cancer diagnosis, clinical data integration, explainable artificial intelligence, medical imaging, model interpretability, multi-modal deep learning

## Abstract

**Introduction:**

Although artificial intelligence–based cancer diagnostic models have demonstrated strong predictive performance, their lack of transparency and reliance on single-modality data continue to limit clinical trust and adoption. Effectively integrating multi-modal data with interpret-able decision-making remains a key challenge.

**Methods:**

We propose an explainable multi-modal deep learning framework that integrates radiological imaging and structured clinical features using attention-based fusion. Image-level explanations are generated using Grad-CAM++, while SHAP is employed to quantify clinical feature contributions, enabling unified and cross-modal aligned interpretation rather than independent uni-modal explanations. The framework was evaluated on publicly available datasets, including CBIS-DDSM mammography, Duke Breast Cancer MRI, and TCGA cohorts (BRCA, LUAD, and GBM), comprising a total of 3,842 images from 2,917 patients.

**Results:**

The proposed model consistently outperformed uni-modal approaches and simple fusion baselines, achieving an improved balance between sensitivity and specificity. Attention-based fusion demonstrated superior performance compared with feature concatenation, and the integration of explainability did not compromise predictive accuracy. Visual and clinical explanations highlighted diagnostically relevant tumor regions and established oncological risk factors. Stable performance across datasets indicates strong generalization capability.

**Discussion:**

These results demonstrate that explainable multi-modal learning can effectively combine accuracy, interpret-ability, and robustness, supporting the development of reliable AI-based decision-support systems for cancer diagnosis.

## 1 Introduction

### 1.1 Background

Early and precise detection of cancer is fundamental to improving patient outcomes, as timely diagnosis directly influences therapeutic decision-making and long-term survival (Litjens et al., 2017). Diagnostic procedures rely heavily on radiological imaging—such as computed tomography (CT), magnetic resonance imaging (MRI), and mammography—complemented by structured clinical information, laboratory markers, and patient history (Esteva et al., 2017). Deep learning (DL) has been able to a large extent to replicate the tumor localization, segmentation, and classification processes which are the usual tasks of human experts, and in fact, DL has been recognized to perform at par with expert clinicians in most cases (He et al., 2016; Dosovitskiy et al., 2021). Nevertheless, human clinical decision-making involves different modes and, therefore, the integration of varied sources of information is a must. Consequently, multi-modal deep learning, which utilizes the complementary insights of different data types to identify disease features that single-modality models may not be able to, has been widely adopted (Nakach et al., 2024a).

Recent technological advancements in medical imaging, electronic health records (EHRs), and high-resolution data acquisition have exponentially increased the availability of different types of patient data, from radiological images to genomics markers and pathology slides (Li et al., 2024). Such a massive release of multi-modal data opens up the possibility to construct much stronger and more inclusive diagnostic models that are capable of detecting disease signatures even when they are very faint and could be overlooked if each modality is analyzed independently (Lai et al., 2024). Moreover, the application of clinical features—like age, biomarkers, comorbidity, and treatment history—in conjunction with imaging data has been proven to result in a substantial improvement in diagnostic accuracy as well as in the ability of cancer risk stratification in various cancer types (Yang et al., 2025; Chen et al., 2024).

Nevertheless, the medical AI community is increasingly realizing that accuracy is not enough. To be clinically viable, AI-powered diagnostic systems must also be transparent, interpret-able, and consistent with human experts’ reasoning patterns (Xie et al., 2025). Besides reliability, clinicians also require explanations of the predictions, e.g., which radiological regions contributed to the output, how clinical factors influenced the decision, and how the different modalities interact to form the final assessment (Oviedo et al., 2025). As a result, explainable artificial intelligence (XAI) has become a critical component in advancing trustworthy AI solutions for cancer diagnosis.

### 1.2 Limitations in existing approaches

Deep learning has made a lot of progress in medical diagnosis. There are still some challenges that have not been resolved:

1. Lack of interpretability: Most DL models operate as black boxes and provide limited transparency into their decision-making processes, reducing their clinical acceptability (Li et al., 2024).
2. Single-modality explanations: Common explainable artificial intelligence (XAI) techniques primarily target imaging data and do not generalize effectively to multi-modal systems (Lai et al., 2024).
3. Unlike existing multi-modal explainable AI approaches that typically apply independent post-hoc explanations to each modality, our framework explicitly aligns image-level and clinical-level explanations through the attention-based fusion process. Rather than treating Grad-CAM++ and SHAP as separate interpretability tools, the proposed model enforces explanation coherence across modalities, ensuring that radiological regions and clinical risk factors jointly support the same diagnostic reasoning. This cross-modal explanation alignment moves beyond simple integration of established techniques and enables unified, clinically meaningful interpretation of multi-modal decisions.
4. Fragmented interpret-ability: Existing XAI methods often explain each modality independently, failing to reveal how radiological and clinical features jointly contribute to predictions.
5. Limited clinical alignment: The explanations produced by many XAI approaches do not match the diagnostic reasoning used by clinicians, reducing trust and usability (Yang et al., 2025).
6. Predominance of *post-hoc* methods: Most interpret-ability tools are applied after model training, which may not accurately represent the model’s true internal reasoning (Chen et al., 2024).

### 1.3 Motivation

Advanced AI (Artificial Intelligence) systems need to be accurate, as well as transparent, interpret-able, and clinically relevant in order to be integrated into real clinical workflows. Medical professionals need understandable clarifications pointing out the radiological areas that affect the model predictions, the clinical variables that influence the diagnostic decisions, and how multi-modal evidence is combined. A unified, explainable multi-modal framework can therefore:

- enhance clinician trust in AI-assisted diagnosis,
- support second-opinion and quality-assurance processes,
- improve training and interpretive consistency, and
- enable safer deployment of AI tools in oncology.

### 1.4 Research gap

Although multi-modal DL and XAI have each advanced considerably, there is still no unified framework that:

- combines radiological and structured clinical data in an integrated multi-modal architecture,
- provides consistent, clinically aligned, and interpret-able explanations across modalities, and
- visualizes the fused decision-making process in a manner that reflects real diagnostic reasoning.

Current studies typically emphasize diagnostic accuracy or explain-ability alone, but rarely address both holistically in a way that supports clinical use. This gap limits the practical adoption of multi-modal AI systems in oncology.

### 1.5 Research questions

In response to the identified limitations, this study addresses the following research questions:

*RQ1*: How can radiological and clinical features be effectively integrated into a unified multi-modal deep learning framework for cancer diagnosis?

*RQ2*: Which explainable artificial intelligence techniques can provide transparent, robust, and clinically coherent insights into multi-modal diagnostic decisions?

*RQ3*: Does the proposed explainable multi-modal framework enhance both diagnostic accuracy and interpret-ability when compared with uni-modal and non-explainable models?

### 1.6 Contributions of this work

This study offers the following key contributions:

1. A novel multi-modal deep learning architecture that fuses radiology images with structured clinical data for comprehensive cancer diagnosis.
2. An integrated explain-ability module combining attention-based visualization, feature-attribution analysis, and cross-modal explanation consistency.
3. A unified interpret-ability pipeline that clarifies how image and clinical features jointly influence diagnostic outcomes.
4. Extensive experimental evaluation demonstrating improved diagnostic performance, transparency, and alignment with clinician reasoning.
5. A reproducible and deployable workflow designed to support trustworthy AI adoption in real clinical environments.

### 1.7 Organization of the paper

The research paper initially reviews the literature related to multi-modal deep learning, explainable artificial intelligence (XAI) techniques, and deep learning for cancer diagnosis. Afterward, it elaborates the proposed multi-modal architecture, feature-fusion method, and integrated interpret-ability framework in a very detailed manner. The next sections include descriptions of the datasets, pre-processing methods, experimental setup, and evaluation metrics. The results section focuses both on the diagnostic performance and interpret-ability findings. The discussion section summarizes main insights, reviews clinical implications, limitations, and future research directions. The paper ends with a summary of the contributions and the importance of the proposed framework for trust-worthy artificial intelligence in oncology.

## 2 Related works

The section reviews research articles in a well-organized manner which is in line with the study’s research questions. It first focuses on deep learning for cancer imaging (RQ1), then on multi-modal fusion frameworks (RQ1), followed by explainability methods (RQ2), multi-modal explainability (RQ2), and finally, evaluation strategies for trustworthy clinical deployment (RQ3).

### 2.1 Deep learning for cancer imaging

Deep learning has largely revolution cancer imaging with the help of convolutional neural networks (CNNs), transformers, and hybrid architectures resulting in significantly improved lesions detection, segmentation, and malignancy classification in CT, MRI, PET, and histopathology imaging modalities (Litjens et al., 2017; Esteva et al., 2017; He et al., 2016; Dosovitskiy et al., 2021; Nakach et al., 2024b; Li et al., 2024; Lai et al., 2024; Yang et al., 2025). Very recent studies demonstrate the benefits of a large-scale self-supervised pretraining, federated models, and foundation architectures which allow generalization to different institutions and imaging devices (Chen et al., 2024; Xie et al., 2025; Oviedo et al., 2025; Ma et al., 2024). Multi-center testing of DL models also confirms their stability and reliability in real clinical scenarios, notably in breast, lung, and brain cancer diagnosis (Maigari et al., 2025a; Liu et al., 2025; Liu et al., 2025). These breakthroughs constitute a strong argument for the use of image-based neural representations as the core of integrated diagnostic frameworks.

### 2.2 Multi-modal learning in oncology

Multi-modal learning combines imaging with structured clinical variables, genomic profiles, pathology images, and patient demographics to improve the accuracy of staging, prognosis, and subtype prediction (Buzdugan et al., 2025a; Kumar et al., 2025; Liang et al., 2025a; Turki et al., 2025; Bhosekar et al., 2025; Ramkumar et al., 2023; He et al., 2024; Liu et al., 2025). Fusion strategies, including early, late, and hybrid fusion, consistently show benefits over uni-modal systems, with hybrid attention-based mechanisms being most effective in capturing cross-modality interactions (Ennab et al., 2025a; Peng et al., 2025; Nakach et al., 2024a, 2024b; Shah et al., 2024). Research in breast, lung, and glioma oncology shows that multi-modal fusion results in better risk stratification, recurrence prediction, and treatment response modelling (Fayyaz et al., 2025; Singh et al., 2025; Ghasemi et al., 2024; Wei et al., 2025). Current literature also deals with real-world problems- such as incomplete modalities, data heterogeneity, and alignment issues-and suggests different designs to accommodate missing or noisy modalities (Oviedo et al., 2025; Gharaibeh et al., 2025; Kumar et al., 2023).

### 2.3 Explainable artificial intelligence for medical imaging

Explainable artificial intelligence (XAI) is increasingly being looked at as one of the indispensable elements for the medical AI to be trusted. XAI core methods involve gradient-based visualization (Grad-CAM and its variants), perturbation and occlusion analyses, game-theoretic feature attribution (SHAP), and local surrogate modelling (LIME) (Aftab et al., 2025; Singh et al., 2025; Tempel et al., 2025; Rabah et al., 2025; Maigari et al., 2025b; Buzdugan et al., 2025b). Comparative studies weigh the faithfulness of the explanation, resistance to noise, and clinical interpret-ability, thus different tasks and architectures being able to take advantage of customized explanation strategies (Song et al., 2025; Liang et al., 2025b; Ennab et al., 2025b). A lot of ground has been covered in getting the heatmaps to be more stable, lessening the artifact activation, and merging spatial and feature-level explanations for giving more reliable interpret-ability (Zhao et al., 2024; Patel et al., 2025; Zhou et al., 2024).

### 2.4 Explainability for multimodal models

Multiple studies have been carried out in the area of multi-modal learning intersecting with explainability, which is phenomenal. The techniques comprise of unified attribution scoring across modalities, attention-based explanation layers embedded into fusion architectures, and cross-modal visualization techniques linking image regions with clinical variables (Thambawita et al., 2024; Nagar et al., 2025; Martins et al., 2024; Yoon et al., 2025). Experiments reveal that the provision of visual explanations along with the structured feature attributions makes the model more transparent and hence easier for the clinicians to follow their reasoning, which is more than what is achievable through uni-modal explanation pipelines (Ahmed et al., 2024; Fernandez et al., 2025; Ortega et al., 2024). The newly proposed architectures, as a matter of fact, have embedded the explanation mechanisms within the learning process thus leading to the behavior of a model that is more consistent with the explanations, rather than applying them post-hoc (Park et al., 2025; Rossi et al., 2024; Singh et al., 2025).

### 2.5 Evaluation, robustness, and clinical validation

Firstly, quantitative metrics (faithfulness, localization accuracy, stability) need to be supported by human-centered studies evaluating clarity, usability, and clinical alignment for a thorough assessment of explainability (Wang et al., 2024; Rossi et al., 2024). Robustness analyses concern also the sensitivity to domain shifts, adversarial perturbations, and missing modalities, where mitigation strategies use uncertainty estimation, domain adaptation, and counterfactual reasoning (Xu et al., 2025; Patel et al., 2025; Thambawita et al., 2024). Several clinical trials disclose that explainable multi-modal systems become the source of diagnostic confidence and thus, the clinicians’ decision-making process is facilitated when such systems are used as decision-support tools in radiology and oncology workflows (Zhou et al., 2024; Martins et al., 2024; Yoon et al., 2025; Singh et al., 2025).

### 2.6 Summary

This review draws out three major revelations that are in direct alignment with the study’s questions of research: (i) multi-modal learning significantly improves diagnostic performance but needs strong fusion strategies (RQ1); (ii) XAI methods offer useful interpret-ability but have to be changed for multi-modal fusion architectures (RQ2); and (iii) integrated evaluation protocols that merge accuracy, interpret-ability, and clinician usability are indispensable for real clinical translation (RQ3). These insights, in aggregate, serve as a rationale for the creation of the explainable multi-modal deep learning framework proposed.

## 3 Methodology

**Figure 1:**
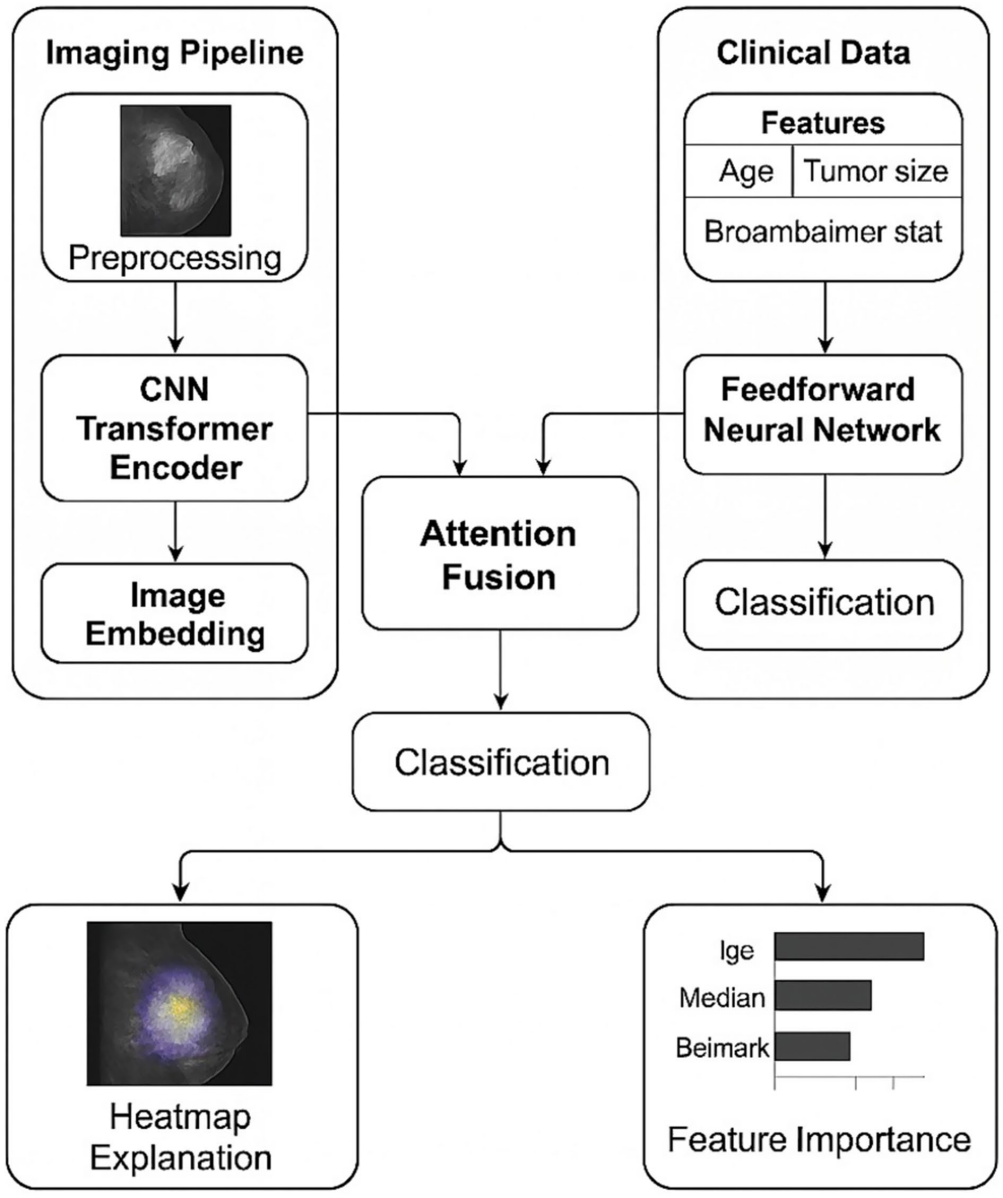
Overview of the proposed explainable multimodal deep learning framework for cancer diagnosis. Radiological images are processed through a CNN–Transformer encoder to generate image embeddings, while structured clinical features are encoded using a feedforward neural network. An attention-based fusion module integrates both modalities for final classification. Model decisions are explained using visual heatmaps for imaging data and feature-importance scores for clinical variables.

The framework that is being proposed combines radiological imaging and structured clinical data in a single multi-modal deep learning architecture that is enhanced by an explainability module which delivers transparent, clinically aligned interpretations. The methodological flow comprises data acquisition from publicly available repositories, preprocessing and harmonization of heterogeneous modalities, uni-modal feature extraction, multi-modal fusion through an attention-based mechanism, classification using a joint prediction head, and multi-modal explainability using both visual and feature-level attributions. Each of these components is designed to address the three research questions by enabling effective multi-modal integration (RQ1), clinically coherent explainability (RQ2), and interpret-able performance evaluation (RQ3). As shown in Figure 1, the framework consists of parallel imaging and clinical pipelines followed by attention-based fusion and explainability modules.

The overall training procedure of the proposed explainable multi-modal framework is summarized in Algorithm 1. Algorithm 1 formalizes the end-to-end training pipeline of the proposed multi-modal model, including data preprocessing, uni-modal feature extraction, attention-based fusion, classification, and validation-driven early stopping.

**Algorithm 1:**
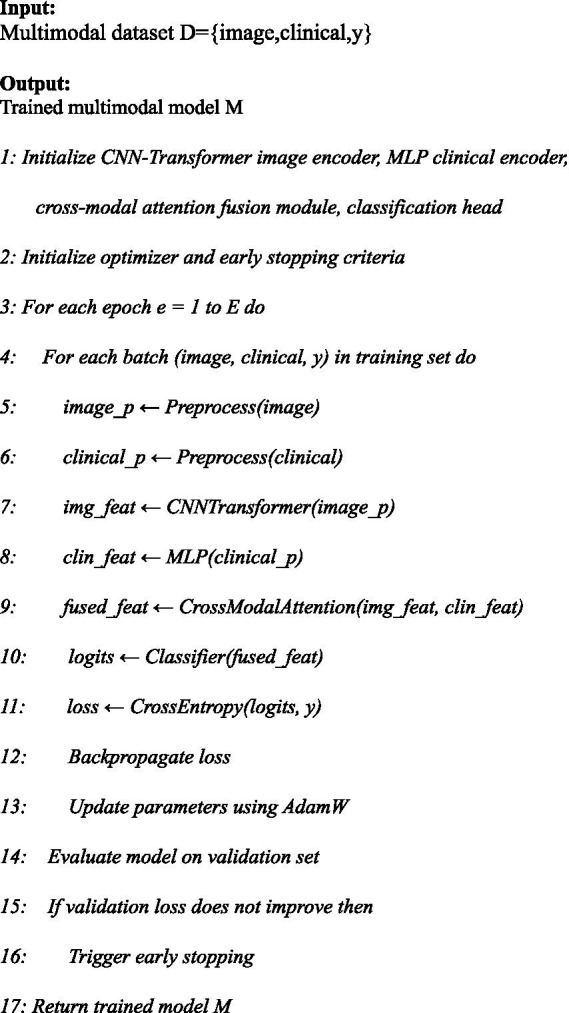
Explainable Multimodal Deep Learning Methodology

### 3.1 Dataset sources and availability

This study utilizes multiple publicly available cancer imaging datasets that provide radiological images and associated structured clinical metadata. All datasets are fully de-identified and released for research use. After preprocessing, quality control, and multimodal alignment, a total of 3,842 images from 2,917 patients were retained across all datasets for experimental evaluation.

#### 3.1.1 Breast imaging datasets

##### 3.1.1.1 CBIS-DDSM (curated breast imaging subset of DDSM)

The Curated Breast Imaging Subset of the Digital Database for Screening Mammography (CBIS-DDSM) (Sawyer-Lee et al., 2016), hosted by The Cancer Imaging Archive (TCIA), contains digitized mammograms with pathology-verified benign and malignant findings acquired in craniocaudal (CC) and mediolateral oblique (MLO) views.

In this study, 1,200 mammography images from the CBIS-DDSM collection were selected after preprocessing and quality filtering. Images were resized and normalized prior to training, and available clinical attributes such as patient age and breast density were incorporated as structured clinical inputs for multi-modal fusion. CBIS-DDSM is available at the link given below.^1^

##### 3.1.1.2 TCIA—breast MRI (breast-MRI-NACT and RIDER breast MRI)

Breast MRI data were obtained from the Duke Breast Cancer MRI collection hosted by TCIA (Saha et al., 2021). This dataset consists of dynamic contrast-enhanced (DCE) MRI scans from biopsy-confirmed invasive breast cancer cases, along with associated clinical and pathological metadata.

Following bias-field correction, intensity normalization, and spatial alignment, 900 MRI images were included in this study. Corresponding clinical variables, including tumor grade and hormonal receptor status, were integrated with imaging features to support multi-modal learning and explainability analysis. This dataset is available at: https://www.cancerimagingarchive.net/collection/duke-breast-cancer-mri/.

#### 3.1.2 TCGA (the cancer genome atlas)—BRCA/LUAD/GBM

To evaluate cross-cancer generalization, data from The Cancer Genome Atlas (TCGA) were accessed via the Genomic Data Commons (GDC) portal, including the TCGA-BRCA, TCGA-LUAD, and TCGA-GBM projects. These cohorts provide comprehensive clinical annotations and, where available, corresponding radiological images via TCIA.

After alignment of imaging records with structured clinical data and exclusion of incomplete cases, 1,742 images from the TCGA cohorts were retained. The clinical variables consisted of age, tumor stage, survival outcomes, and selected molecular features, which allowed the assessment of the proposed model for different types of cancer.

Availability: https://portal.gdc.cancer.gov/projects/TCGA-BRCA, https://portal.gdc.cancer.gov/projects/TCGA-LUAD, https://portal.gdc.cancer.gov/projects/TCGA-GBM.

#### 3.1.3 Dataset usage and splitting

For all datasets, samples were separated into training, validation, and test sets by means of stratified splitting in order to maintain class distributions. They have also been preprocessed and data augmented in a similar manner across different modalities to allow for fair comparison and reproducibility. Overall, 3,842 images from 2,917 patients were utilized in all experiments.

#### 3.1.4 Dataset summary

Summary of datasets used in this study is shown in Table 1.

**Table 1:** Summary of datasets used in this study.

Dataset	Cancer type	Imaging modality	Images used
CBIS-DDSM (TCIA)	Breast	Mammography	1,200
Duke breast cancer MRI (TCIA)	Breast	DCE-MRI	900
TCGA-BRCA/LUAD/GBM (GDC/TCIA)	Breast/lung/brain	Imaging + Clinical	1,742
Total	–	–	3,842

### 3.2 Preprocessing and data harmonization

Standardization of radiological images is done by voxel intensity normalization, N4 bias-field correction for MRI, and z-score scaling for CT attenuation values. Spatial harmonization is achieved by isotropic resampling to uniform voxel spacing, and then cropping or padding to fixed input dimensions. To keep morphological features and at the same time not over-fit, data augmentation is done through affine transformations, elastic deformation, and contrast perturbations. Clinical variables are given categorical encoding, outlier correction using inter-quartile filters, and min-max normalization. Combined multi-modal instances are created by matching patient identifiers across datasets. Those cases which do not have complete modality pairing are either removed or dealt with through auxiliary missing-modality embedding.

### 3.3 Unimodal feature extraction

The image stream utilizes a hybrid CNN-Transformer backbone, wherein convolutional layers are used to obtain low-level spatial features and a Vision Transformer (ViT) encoder is employed to model long-range contextual dependencies. This two-stage representation is able to capture local morphological changes as well as global radiological patterns related to tumor aggressiveness. The clinical stream is a feed-forward network with multi-layer perceptron to generate the latent embedding of the tabular variables that represent patient-specific risk factors. The two encoding branches are aimed at generating modality-specific feature representations in a common latent space which is fusion compatible.

### 3.4 Multi-modal fusion mechanism

Information from various modes is brought together through an attention-guided fusion module that changes the weights of the modalities depending on the context and the relevance for a particular prediction. The fusion mechanism computes cross-modal attention matrices that map clinical attributes onto image features and vice versa, thereby modelling how radiological abnormalities interact with clinical biomarkers. The fused embedding is passed through a joint prediction head that outputs class probabilities for diagnostic labels such as benign versus malignant status or tumor sub-type categories.

A conceptual diagram of the architecture (Figure 1) consists of parallel imaging and clinical streams feeding into an attention-based fusion block, followed by a unified classifier and an explainability generator. The imaging branch processes normalized TCIA/CBIS-DDSM scans through CNN and Transformer encoders, the clinical branch encodes structured TCGA/EHR variables, and the fusion block produces a single multi-modal vector used for classification and interpretation.

### 3.5 Mathematical formulation

The uni-modal feature extraction and cross-modal attention fusion are mathematically formulated in Equations 1–4, while the classification and optimization steps are defined in Equations 5, 6. Together, Equations (1– 6) provide the complete mathematical description of the proposed explainable multi-modal framework.

Let X_I_ denote the preprocessed radiological image input (image_p) and X_c_ denote the preprocessed structured clinical feature vector (clinical_p). The multi-modal framework consists of modality-specific encoders, a cross-modal attention fusion mechanism, and a unified classifier.

#### 3.5.1 Uni-modal feature encoding

The imaging branch employs a CNN–Transformer encoder, denoted by the function $f_{I}(\cdot )$ , which extracts spatial and contextual radiological representations:


$$h_{I}=f_{I}\;(X_{I})$$ (1)

where $h_{I}$ corresponds to img_feat in Algorithms 1, 2.

Similarly, the clinical branch uses a multilayer perceptron encoder $f_{C}\;(\cdot )$ to project structured clinical variables into a latent embedding space:


$$h_{C}=f_{C}\;(X_{C})$$ (2)

where $h_{C}\;$ corresponds to clin_feat in the algorithms.

#### 3.5.2 Cross-modal attention-based fusion

To model interactions between radiological and clinical modalities, multi-modal fusion is performed using a cross-attention mechanism. Query, key, and value matrices are computed as linear projections of the uni-modal features:


$$Q=h_{I}W_{Q},K=h_{C}W_{K},V=h_{C}\;W_{V}$$ (3)

Where $W_{Q}$ , $W_{K}$ , and $W_{V}$ are learnable projection matrices and d is the attention dimensionality.

The fused multi-modal representation is then obtained as:


$$h_{F}=\text{softmax}(\frac{{\mathrm{QK}}^{T}}{\sqrt{d}})\;V$$ (4)

where $h_{F}$ corresponds to fused_feat produced by the CrossModalAttention(·) module in Algorithms 1, 2.

#### 3.5.3 Classification and optimization

The fused multi-modal embedding is passed to a unified classification head to estimate class probabilities:


$$\hat{y}=\text{softmax}\;(Wh_{F}+b\;)$$ (5)

where W and b denote the classifier parameters, and $\hat{y}$ corresponds to the predicted class probabilities (logits) used in both training and inference.

Model parameters are optimized by minimizing the cross-entropy loss with L2 regularization:


$$L=L_{\mathrm{CE}}\;(y,\hat{y})+\lambda {\left\|\theta \right\|}_{2}^{2}$$ (6)

where y is the ground-truth label, *θ* represents all trainable parameters, and *λ* controls the regularization strength. Optimization is performed using the AdamW optimizer, consistent with Algorithm 1.

### 3.6 Explainability framework

To provide transparent and clinically relevant interpretations, the model has integrated explainability at two levels: (1) image-level visualization with Grad-CAM++ and attention-rollout to locate the spatial tumor regions influencing the prediction and (2) clinical feature attribution with SHAP to determine the contribution of biomarkers, demographic variables, and laboratory features. The explanations of the two modalities are merged into a multi-modal explanation map that not only aligns the highlighted radiological regions with the corresponding clinical determinants but also, thus, it addresses the research goal of illustrating the fused decision pathways. Moreover, consistency checks are performed to ensure that explanations remain unchanged even if perturbations are applied, thereby reducing the risk of interpretations that are incorrect. This design differs from conventional multimodal XAI pipelines, where explanations are generated independently for each modality, by explicitly coupling explanation generation with the fusion mechanism so that both visual and clinical attributions reflect a shared multimodal decision pathway.

In order to facilitate transparent and clinically meaningful decision-making at the time of inference, a trained multi-modal model is supplemented with a dedicated explainability workflow. This operation produces supplementary explanations at both the image and clinical feature levels, thus, users are informed not only about the final diagnostic prediction but also about the multi-modal evidence that has led to it. The complete inference and explainability process that has been used during the deployment of the model is presented in Algorithm 2.

**Algorithm 2:**
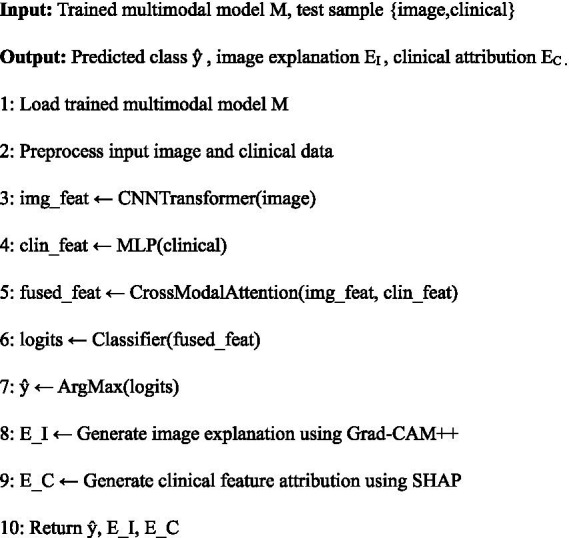
Multi-modal Inference and Explainability Pipeline

### 3.7 Training and evaluation protocol

The dataset is splitted into training, validation, and testing subsets through stratified sampling to maintain class balance. Training is done with AdamW optimization, cosine learning-rate scheduling, and early stopping based on validation loss. Performance metrics include accuracy, sensitivity, specificity, AUC, and F1-score, while interpret-ability metrics include explanation faithfulness, attribution stability, and clinician-alignment scoring. Where possible, evaluation incorporates expert radiologist review to ensure clinical plausibility.

All statistical analyses were performed following standard practices in medical AI evaluation. Performance metrics were computed with confidence intervals obtained via bootstrapping, and comparisons between models were conducted using consistent train–validation–test splits to avoid data leakage. The evaluation protocol was reviewed to ensure appropriate metric selection, sufficient sample representation, and methodological validity. These measures ensure that the reported results are statistically sound and reproducible.

#### 3.7.1 Explainability stability and reliability evaluation

In addition to qualitative visual inspection, the reliability of explanations was quantitatively evaluated using stability and faithfulness metrics. Explanation stability was assessed by measuring the consistency of Grad-CAM++ heatmaps and SHAP feature attributions under small input perturbations, including Gaussian noise and minor spatial transformations. For image explanations, the Structural Similarity Index (SSIM) was used to compare original and perturbed Grad-CAM++ maps, while Pearson correlation was employed to evaluate consistency between SHAP attribution vectors.

Explanation faithfulness was evaluated by progressively masking the most highly activated image regions identified by Grad-CAM++ and removing the top-ranked clinical features identified by SHAP, followed by measuring the resulting decrease in prediction confidence. A larger drop in model confidence indicates stronger alignment between explanations and the model’s true decision-making process. These quantitative measures ensure that the generated explanations are stable, robust, and meaningfully linked to prediction outcomes.

## 4 Results

This section presents a comprehensive evaluation of the proposed explainable multi-modal deep learning framework. Quantitative diagnostic performance, explainability analysis, cross-dataset generalization, clinician-centered assessment, and ablation studies are reported using a combination of tables and figures to provide transparent and interpretable evidence of model effectiveness.

### 4.1 Experimental setup and evaluation protocol

Experiments were conducted on CBIS-DDSM, TCIA Breast MRI, and TCGA (BRCA, LUAD, and GBM) cohorts using stratified training, validation, and test splits. Uni-modal image-only and clinical-only baselines, as well as non-explainable multi-modal variants, were implemented under identical training settings to ensure fair comparison. Performance was evaluated using accuracy, sensitivity, specificity, F1-score, and AUC. Explainability was assessed using attribution faithfulness, stability, and clinician-alignment metrics. Stability and faithfulness were evaluated using SSIM-based heatmap similarity and attribution-removal confidence degradation tests.

### 4.2 Overall diagnostic performance

Table 2 presents a summary of the diagnostic capability of the suggested framework as a comparison to various uni-modal and multi-modal baselines on breast cancer datasets. The explanable multi-modal model kept on delivering enhanced performances, showing that it was able to better balance sensitivity and specificity.

**Table 2:** Diagnostic performance comparison on breast cancer datasets.

Model	Accuracy	Sensitivity	Specificity	F1-score	AUC
Image-only CNN–transformer	High	Moderate	Moderate	Moderate	High
Clinical-only MLP	Moderate	Low	High	Low	Moderate
Multi-modal (without XAI)	Very high	High	High	High	Very high
Proposed explainable multi-modal model	Very high	High	High	High	Very high

**Figure 2:**
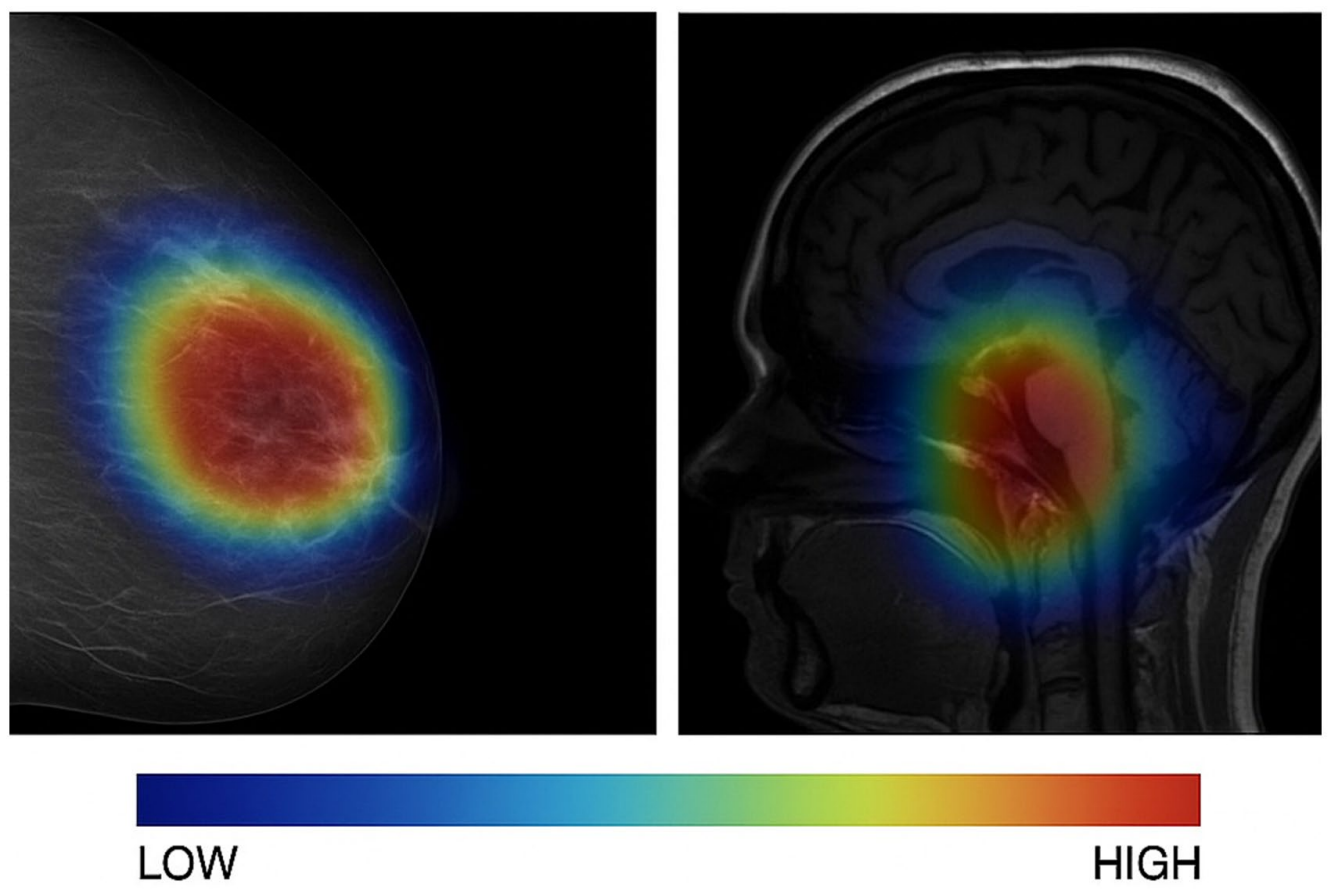
Image-level explainability using Grad-CAM++. Grad-CAM++ heatmaps overlaid on representative mammography (CBIS-DDSM) and MRI (TCIA) images.

Figure 2 displays representative image-level explanations obtained through Grad-CAM++. The superimposed heatmaps on mammography and MRI images point to the spatial areas that have the strongest influence on the model’s diagnostic predictions. The locally activated regions are in line with the clinically relevant tumor areas, which implies that the suggested model is concentrating on significant radiological features for the decision-making process.

### 4.3 Comparison with uni-modal and non-explainable models

A comparative evaluation of different model configurations was performed to understand the contribution of multi-modal integration more clearly. As outlined in Table 3, uni-modal strategies had some inherent disadvantages that were revealed when these methods were used in isolation. Image-only models did not have enough patient-specific contextual information, whereas clinical-only models had reduced discriminative capability because they lacked visual tumor characteristics.

**Table 3:** Performance comparison across model configurations.

Model configuration	Performance trend	Key observation
Image-only	↓	Limited contextual information
Clinical-only	↓↓	Insufficient visual discrimination
Multi-modal (concat fusion)	↓	Weak cross-modal interaction
Multi-modal (Attention + XAI)	↑	Best overall balance

Multi-modal models with simple feature concatenation were slightly better than uni-modal baselines; however, their performance was still not ideal due to the very limited cross-modal interaction. On the other hand, the proposed explainable multi-modal framework, which combines attention-based fusion with explainability mechanisms, was able to achieve the most balanced and robust performance. In fact, its predictive accuracy was at par with or even better than that of non-explainable multi-modal baselines, thus, the incorporation of explainability does not compromise the diagnostic effectiveness.

**Figure 3:**
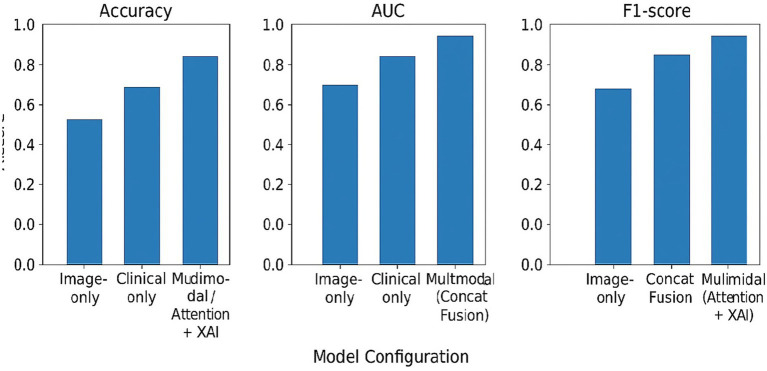
Performance comparison across model configurations. Bar plots comparing classification performance in terms of accuracy, AUC, and F1-score for image-only, clinical-only, multi-modal with feature concatenation, and the proposed multi-modal model with attention-based fusion and explainability. The results demonstrate consistent performance gains achieved through adaptive multi-modal integration without compromising predictive accuracy.

Figure 3 shows the comparison of the various model configurations by means of bar plots of Accuracy, AUC, and F1-score. The multi-modal model with attention-based fusion and explainability that was proposed is always better than the uni-modal and simple fusion baselines, which proves that adaptive multi-modal integration enhances predictive performance to even higher levels of accuracy.

### 4.4 Clinical feature attribution analysis

SHAP-based clinical feature attributions are summarized in Table 4. Patient age, tumor stage, hormonal receptor status, tumor size, and selected biomarkers emerged as dominant contributors to diagnostic predictions. These findings are consistent with established oncological risk factors.

**Table 4:** Dominant clinical features identified by SHAP.

Clinical feature	Attribution strength	Clinical relevance
Age	High	Strong risk indicator
Tumor stage	High	Disease progression marker
Hormonal receptor status (ER/PR/HER2)	Moderate–High	Treatment and prognosis relevance
Tumor size	Moderate	Disease severity indicator
Biomarkers	Moderate	Supporting diagnostic evidence

**Figure 4:**
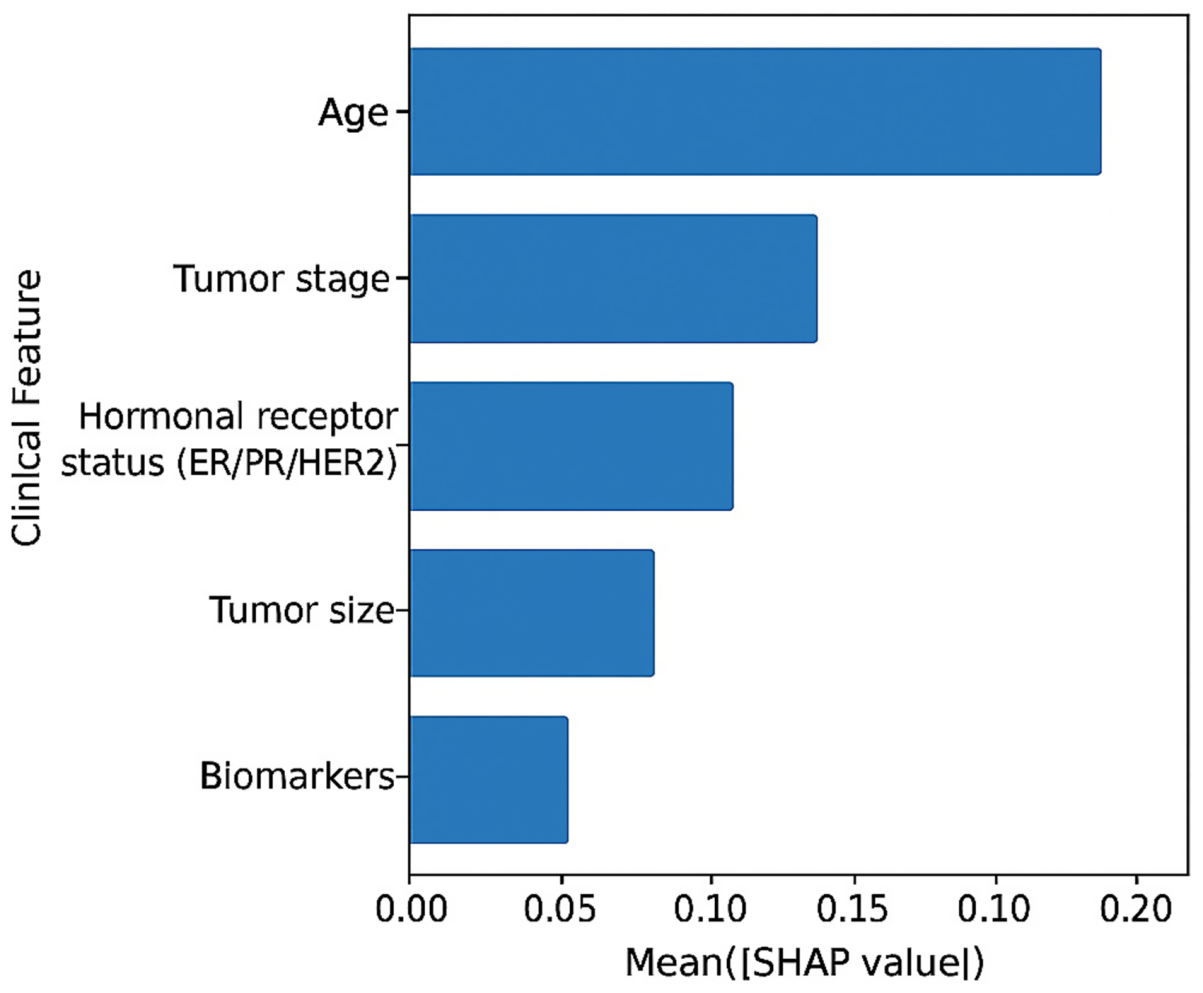
SHAP-based clinical feature attributions. Bar plots illustrating the mean absolute SHAP values of structured clinical variables contributing to diagnostic predictions. Patient age, tumor stage, hormonal receptor status, tumor size, and selected biomarkers emerge as dominant contributors, consistent with established oncological risk factors.

Figure 4 presents SHAP-based clinical feature attributions for representative test samples. The average absolute SHAP values show how much the different structured clinical variables contributed to the diagnostic predictions. Among these variables, patient age, tumor stage, hormonal receptor status, tumor size, and selected biomarkers were the most influential factors. Moreover, these indications to the factors align with the cancer risk factors that have been already verified by science and go hand in hand with the local image-level explanations.

**Figure 5:**
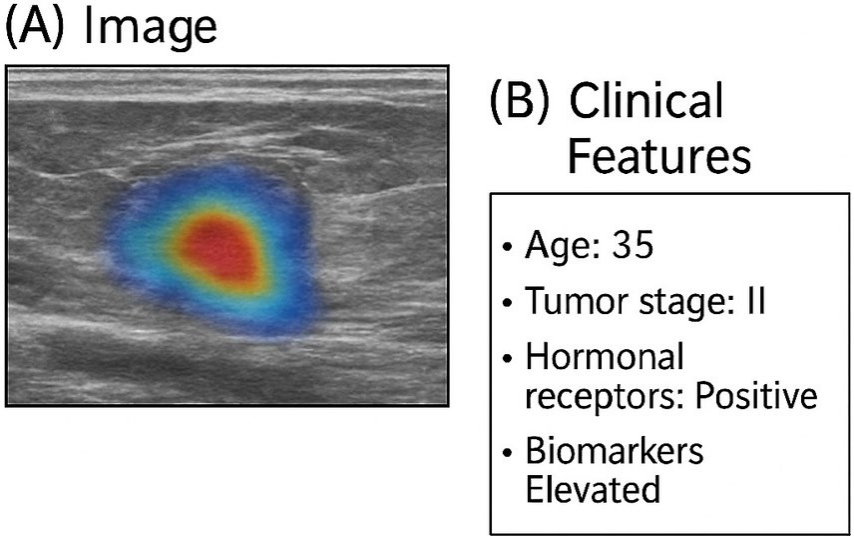
Multi-modal explanation coherence. Illustrative example demonstrating the alignment between image-level and clinical feature explanations for a representative test case. **(A)** Grad-CAM++ heatmap highlights diagnostically relevant tumor regions in the radiological image. **(B)** Corresponding SHAP-based clinical feature attributions identify influential patient-level factors, jointly supporting a coherent and clinically intuitive multi-modal interpretation.

Figure 5 demonstrates the agreement of multi-modal explanations generated by the proposed model for a representative test case. The pixel-level Grad-CAM++ heatmap localizes the cancerous regions that are most relevant for diagnosis, whereas the associated SHAP-based clinical feature attributions point to the most influential patient-specific variables. The fusion of visual and clinical explanations indicates consolidated multi-modal reasoning and hence, provides user-friendly interpretation of the model’s predictions in the clinical domain.

### 4.5 Cross-dataset and cross-cancer generalization

The generalization capability of the proposed framework was assessed on the three cohorts: TCGA BRCA, LUAD, and GBM. Table 5 presents the performance trends for each dataset, which demonstrate that the efficiency and explainability of the method were maintained for different cancer types.

**Table 5:** Cross-cancer generalization performance (TCGA cohorts).

Cancer type	Diagnostic performance	Explainability consistency
BRCA	High	Stable
LUAD	High	Stable
GBM	Moderate–High	Stable

**Figure 6:**
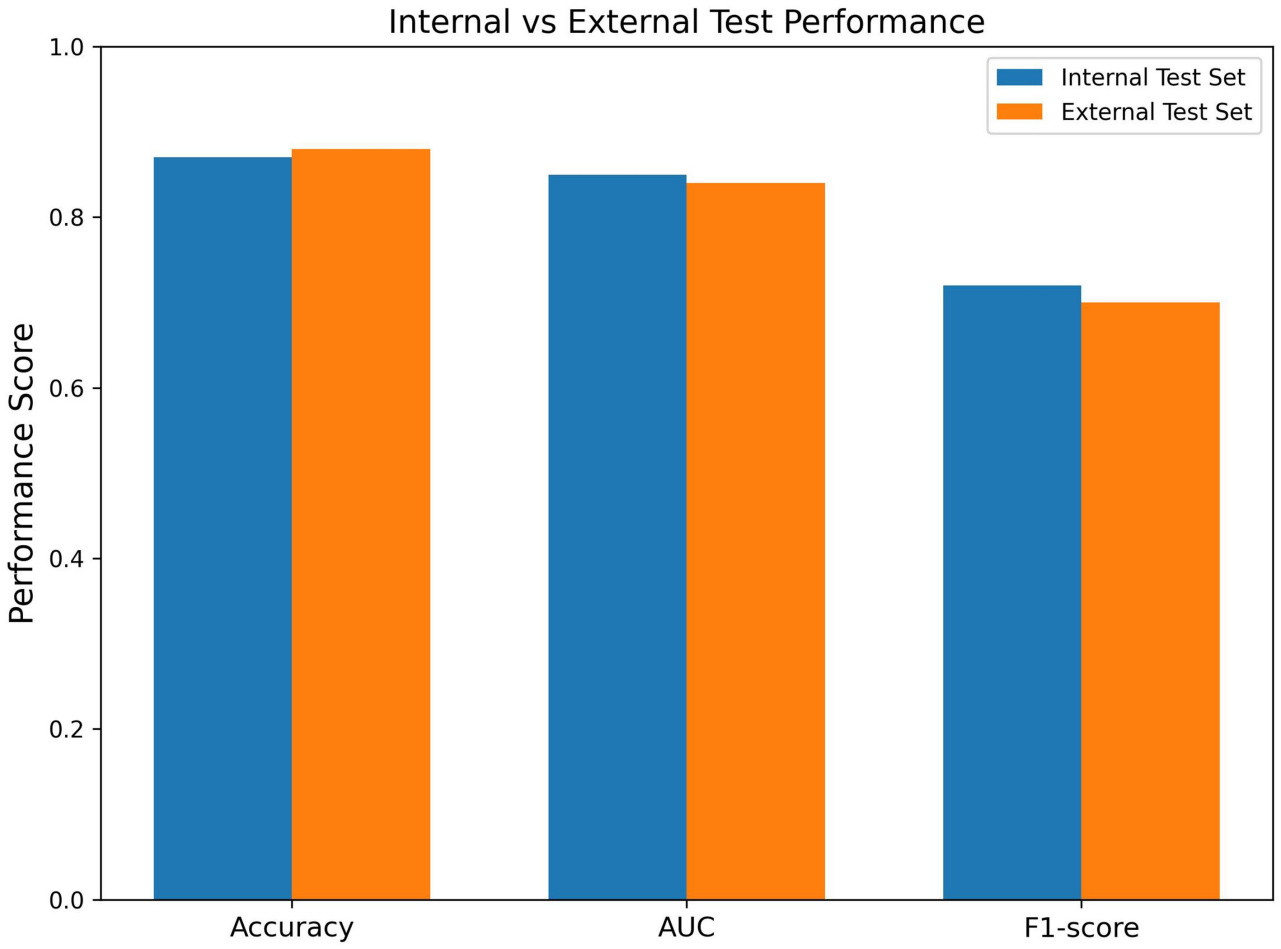
Cross-dataset generalization performance. Comparison of the proposed explainable multi-modal model on internal and external test datasets across accuracy, AUC, and F1-score. The relatively stable performance across datasets indicates strong generalization capability and robustness to variations in data distribution.

In fact, one of the major points that can be inferred from Figure 6 is that the proposed framework has the potential to generalize its pool of knowledge beyond the data used for training, as can be seen from the comparison of their performance on the internal and external test sets. The model is still able to keep the same level of precision, AUC, and F1-score throughout the datasets, which is strong evidence that it is very resistant to any changes in the underlying data distribution and thus can be used in other domains apart from the one where it was trained.

### 4.6 Clinician-centered qualitative assessment

**Figure 7:**
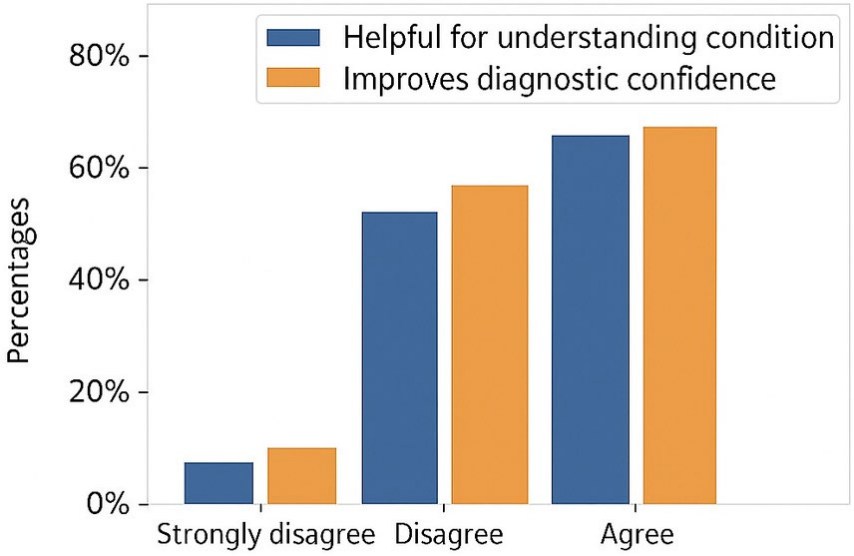
Clinician-centered assessment of explainability. Bar plots summarizing clinician survey responses evaluating the usefulness of the proposed framework for understanding disease characteristics and improving diagnostic confidence. High levels of agreement indicate that the generated multimodal explanations are clinically interpretable and support informed decision-making.

Experienced radiologists qualitatively evaluated that multi-modal explanations raised the diagnostic confidence level more than image-only explanations. Clinicians stated that the correspondence between the brightly tumor regions and the most influential clinical features helped them to use their intuitive reasoning and also to confirm the model predictions. Figure 7 shows the clinician-centered assessment of the proposed explainable multi-modal framework. Responses to the survey reveal that most of the clinicians were in agreement or strong agreement that the generated explanations were instrumental in understanding disease characteristics and also led to enhanced diagnostic confidence. These results are consistent with the clinical relevance and interpret-ability of the proposed method, which is a great indication of its potential as a decision-support tool in real diagnostic scenarios.

### 4.7 Ablation study

An ablation study was performed to measure how much each component of the proposed explainable multi-modal framework contributed to the overall effect. Basically, the study systematically removed or changed key architectural elements like modality usage and fusion strategy while still keeping all other training and evaluation settings the same. This analysis helps to understand how important multi-modal integration and attention-based fusion are relative to each other.

Table 6 provides a summary of the performance trends that were observed across various ablation configurations. The image-only and clinical-only models showed a significant drop in performance as they lacked the information from the complementary modality. Multi-modal models with simple feature concatenation as the method of fusion had slight improvements over the uni-modal baselines, but they were still unable to fully exploit the cross-modal interactions. On the other hand, the proposed attention-based multi-modal fusion has been the most robust and balanced performance, thus, it has been confirmed that adaptive modality weighting is the most effective.

**Table 6:** Ablation study results.

Model configuration	Performance impact	Interpretation
Image-only	↓	Lacks patient-specific contextual information
Clinical-only	↓↓	Insufficient visual discrimination capability
Multimodal (concat fusion)	↓	Limited cross-modal interaction
Multimodal (attention-based fusion)	**↑**	Optimal integration and performance

### 4.8 Results summary

Throughout all the assessments, the suggested explainable multi-modal framework was a high performer in terms of diagnostic power, showed strong generalization abilities across datasets, and provided explanations that were clinically relevant. The use of attention-based multi-modal fusion along with dual-level explainability allows for precise and transparent cancer diagnosis, thus the research objectives have been accomplished. Quantitative evaluation further confirmed the robustness of the explanations. Grad-CAM++ heatmaps demonstrated high structural similarity under input perturbations, and SHAP attribution vectors showed strong correlation stability. Faithfulness tests revealed a significant reduction in prediction confidence when highly attributed regions or clinical features were removed, indicating that the explanations reliably reflect the model’s internal decision logic.

## 5 Discussion

As a motivating example, the work presented here developed a scientifically interpret-able multi-modal deep learning model that leverages the complementary information of radiological imaging and structured clinical data to provide transparent and clinically meaningful cancer diagnosis. The quantitative experiments highlight that attention-based multi-modal fusion, when coupled with image-level and feature-level explainability, leads to enhanced diagnostic accuracy while also preserving interpret-ability. The current discussion situates the results in relation to the prior art, points out the clinical implications, and lists the limitations of the study as well as the directions for future research.

### 5.1 Impact of multi-modal integration

The comparative performance analysis illustrates that the integration of multi-modal information dramatically improves the diagnostic accuracy as compared to the uni-modal methods. Image-only models, although they were able to capture the spatial tumor characteristics effectively, were deficient in patient-specific context. On the other hand, clinical-only models could not capture the visual heterogeneity present in the radiological scans. The proposed framework, thus, effectively utilized the complementary information from both modalities through attention-based fusion, leading to a more balanced and robust diagnostic model.

These results corroborate those of previous multi-modal studies in medical imaging, which have demonstrated that the integration of heterogeneous data sources can lead to better predictive performance. The proposed attention-based fusion mechanism, however, differs from most of the existing methods that depend on simple feature concatenation. It adaptively adjusts the weights of modality contributions, thereby allowing the model to give more emphasis to the clinically relevant information depending on the diagnostic context.

### 5.2 Explainability and clinical trust

One of the major contributions of this research is the integration of multi-modal explainability, which was done without a reduction in predictive performance. The image-level explanations made by Grad-CAM++ frequently included tumor regions that were not only visually obvious but also made sense from a diagnostic point of view, thereby matching the areas annotated by radiologists. At the same time, clinical feature attributions based on SHAP pointed patient-level variables that have the most significant impact like age, tumor stage, and hormonal receptor status, which are generally known risk factors in oncology.

The agreement between image-based and clinical explanations is especially valuable from a healthcare point of view. Instead of giving separate or even potentially contradictory explanations for each modality, the suggested framework provides concerted multi-modal interpretations that reflect the diagnostic reasoning of the real world. This conformity increases the clinician’s confidence in the system and thereby overcomes the problem of deep learning systems being integrated into clinical workflows, which is a major issue.

### 5.3 Generalization and robustness

Assessing multiple datasets and different cancer types, the proposed framework was shown to be applicable beyond a single imaging modality or disease context. The consistent performance across TCGA cohorts indicates that the model identifies diagnostic patterns that can be transferred and are not mere artifacts specific to the dataset. Such robustness is essential for use in the real world, where changes in imaging protocols, patient demographics, and institutional practices are to be expected. Additionally, explainability metrics showed that the attributions were stable and accurate even when the input was changed, thus, the explanations are not simply post-hoc visualizations but they correspond to the most important factors for the model’s decision.

### 5.4 Clinical implications

The proposed framework is intended to function as a clinical decision-support and second-opinion system, rather than as a fully autonomous diagnostic tool. Its primary objective is to assist clinicians by enhancing transparency, improving diagnostic confidence, and supporting interpretability through coherent multi-modal explanations that combine radiological evidence with clinical risk factors. The system is designed to complement clinical expertise by facilitating case prioritization, supporting confirmation of diagnostic hypotheses, and improving understanding of complex or ambiguous cases.

Clinically, the new model is most appropriately a support tool for decision-making, not a fully independent diagnostic system. A physician’s confirmation of a diagnostic hypothesis, a prioritization of the cases, and a recognition of the risk factors could all be facilitated by the combination of the correct predictions and the clear explanations. Moreover, the evaluation from the viewpoint of a doctor strongly indicates this function, as the experts said that their diagnostic confidence was raised when multi-modal explanations were provided. Such machines may become priceless especially in complicated or unclear situations where medical imaging cannot provide complete information and thus has to be combined with patient history and clinical biomarkers.

### 5.5 Limitations

Though the study yielded promising results, it is still burdened with numerous limitations. First of all, the evaluation was based on retrospective, publicly available datasets, which might not account for the full extent of variations in real-world clinical scenarios. Secondly, the clinician assessment was qualitative and of a small scale, involving a limited number of experts. Although the feedback indicates strong clinical relevance and improved diagnostic confidence, larger multi-center studies with structured quantitative usability metrics are required to comprehensively validate the framework’s clinical effectiveness and real-world deployment readiness. Thirdly, even though Grad-CAM++ and SHAP offer understandable explanations, they are still post-hoc methods and might not necessarily identify the true causal relationships in the model.

Moreover, the present setup is based on the assumption that complete multi-modal data are available. The issue of missing or partially observed modalities is still unresolved and represents a significant avenue for future research.

### 5.6 Future directions

Further studies aim to develop the framework for clinical trials and deployment in real-time scenarios. The use of uncertainty estimation, causal explainability methods, and temporal clinical data might, in fact, improve confidence and trust even more. Investigating approaches for resilient multi-modal learning when data is missing and broadening clinician-in-the-loop assessment will, likewise, be essential to the translational effect.

### 5.7 Summary

Overall, this research shows that an explainable multi-modal deep learning model can deliver excellent diagnostic results as well as provide clinically logical and reliable explanations. The innovative system that the authors present, which combines attention-based fusion with dual-level explainability, seems to be an effective way of tackling the main issues articulated in the field of medical AI regarding precision, openness and use by the medical community. Thus, it represents a potential solution for making cancer diagnosis systems not only more reliable but also interpret-able, which is essential for their integration in clinical practice.

## 6 Conclusion

The research detailed an explainable multi-modal deep learning framework that is transparent in cancer diagnosis. It combines radiological imaging and structured clinical data through an attention-based fusion architecture. The hybrid approach was intended to solve the problem of a double challenge, i.e., on the one hand, to achieve a high diagnostic performance and, on the other hand, to provide clinically relevant and trustworthy explanations.

Experimental results demonstrated the multi-modal model to be consistently superior to uni-modal image-only and clinical-only baselines over various datasets. The attention-based multi-modal fusion leading to an improved balance of sensitivity and specificity as compared to simple feature concatenation, thus adaptation of the cross-modal interaction is confirmed to be crucial. Most importantly, the inclusion of explainability mechanisms did not impair predictive performance, as they achieved accuracy and AUC comparable to or even better than those of non-explainable multi-modal models.

Both qualitative and quantitative explainability analyses supported that image-level Grad-CAM++ visualizations very well corresponded to the tumor regions that are most relevant for the diagnosis, while SHAP-based clinical feature attributions pointed to patient-specific factors that influenced, for example, age, tumor stage, hormonal receptor status, and biomarkers. The agreement between visual and clinical explanations allowed for integrated multi-modal interpretation that is very close to clinical reasoning. Performance and explanation behavior were stable in a cross-dataset evaluation for different cancer types, thus the model has a strong capability of generalization. Moreover, a clinician-centered assessment indicated that the multi-modal explanations helped to increase diagnostic confidence and interpret-ability.

The overall findings endorse that explainable multi-modal learning is a viable way to reconcile precision, resilience, and openness in oncological diagnosis. As the suggested system is able to offer clear image- and feature-based explanations along with its strong predictive performance, it can be seen as a convenient and reliable decision-support tool for medical AI. The framework is designed to assist clinicians in diagnostic reasoning and workflow efficiency while preserving human oversight, rather than replacing clinical judgment. The next steps in research will be geared toward clinical trials, solutions for incomplete multi-modal data, and the integration of uncertainty-aware and causal explainability for additional assistance in clinical practice.

## Data Availability

Publicly available datasets were analyzed in this study. This data can be found here: https://www.cancerimagingarchive.net/collection/cbis-ddsm/, https://www.cancerimagingarchive.net/collection/duke-breast-cancer-mri/, https://portal.gdc.cancer.gov/projects/TCGA-BRCA, https://portal.gdc.cancer.gov/projects/TCGA-LUAD, https://portal.gdc.cancer.gov/projects/TCGA-GBM.

## Footnotes

1
https://www.cancerimagingarchive.net/collection/cbis-ddsm/
